# The Early Food Insecurity Impacts of COVID-19

**DOI:** 10.3390/nu12072096

**Published:** 2020-07-15

**Authors:** Meredith T. Niles, Farryl Bertmann, Emily H. Belarmino, Thomas Wentworth, Erin Biehl, Roni Neff

**Affiliations:** 1 Department of Nutrition and Food Sciences, University of Vermont, 109 Carrigan Drive, Burlington, VT 05405, USA; fbertman@uvm.edu (F.B.); emily-h.morgan@uvm.edu (E.H.B.); thomas.wentworth@uvm.edu (T.W.); 2 Food Systems Program, University of Vermont, 109 Carrigan Drive, Burlington, VT 05405, USA; 3 Gund Institute for Environment, University of Vermont, 210 Colchester Ave, Burlington, VT 05405, USA; 4 Center for a Livable Future, Johns Hopkins Bloomberg School of Public Health, 615 N. Wolfe Street, W7010, Baltimore, MD 21205, USA; ebiehl1@jhu.edu (E.B.); rneff1@jhu.edu (R.N.); 5 Department of Environmental Health & Engineering, Johns Hopkins Bloomberg School of Public Health, Baltimore, MD 21205, USA; 6 Department of Health Policy and Management, Johns Hopkins Bloomberg School of Public Health, Baltimore, MD 21205, USA

**Keywords:** COVID-19, food security, food access, malnutrition, employment, coronavirus

## Abstract

COVID-19 has disrupted food access and impacted food insecurity, which is associated with numerous adverse individual and public health outcomes. To assess these challenges and understand their impact on food security, we conducted a statewide population-level survey using a convenience sample in Vermont from 29 March to 12 April 2020, during the beginning of a statewide stay-at-home order. We utilized the United States Department of Agriculture six-item validated food security module to measure food insecurity before COVID-19 and since COVID-19. We assessed food insecurity prevalence and reported food access challenges, coping strategies, and perceived helpful interventions among food secure, consistently food insecure (pre-and post-COVID-19), and newly food insecure (post COVID-19) respondents. Among 3219 respondents, there was nearly a one-third increase (32.3%) in household food insecurity since COVID-19 (*p* < 0.001), with 35.5% of food insecure households classified as newly food insecure. Respondents experiencing a job loss were at higher odds of experiencing food insecurity (OR 3.06; 95% CI, 2.114–0.46). We report multiple physical and economic barriers, as well as concerns related to food access during COVID-19. Respondents experiencing household food insecurity had higher odds of facing access challenges and utilizing coping strategies, including two-thirds of households eating less since COVID-19 (*p* < 0.001). Significant differences in coping strategies were documented between respondents in newly food insecure vs. consistently insecure households. These findings have important potential impacts on individual health, including mental health and malnutrition, as well as on future healthcare costs. We suggest proactive strategies to address food insecurity during this crisis.

## 1. Introduction

The global COVID-19 pandemic, and social distancing efforts implemented to slow its spread [1], have disrupted economies and food systems globally and locally, with extensive food security ramifications. Food insecurity—the lack of consistent physical, social, and economic access to adequate and nutritious food that meets dietary needs and food preferences [2]—can lead to serious public health consequences. In 2018, 11.1% of American households were considered food insecure at some point in the year, and 4.3% experienced very low food security, characterized by disrupted eating patterns and reduced food intake [3]. Food insecurity is associated with numerous adverse health outcomes, including chronic conditions such as diabetes mellitus, hypertension, coronary heart disease, depression, and mental health challenges and increased risk of mortality [4,5,6,7]. Evidence from the United States (U.S.) and Canada has found, on average, health care use [8,9] and costs [4,5,8,9] to be substantially higher among adults living with food insecurity compared to others.

Food insecurity tracks closely with national and household economic conditions, with trends paralleling unemployment, poverty, and food prices [3,10,11]. Given the unprecedented rise in U.S. unemployment since mid-March 2020 [12], models based on data from the 2007/2008 recession predict significant and rapid increases in food insecurity [13]. Food insecurity is not just a consequence of an inability to afford food, however. The COVID-19 pandemic affects all dimensions of food security, defined by the United Nations to include food availability, accessibility, utilization, and stability [2]. Food availability has shifted in the short term by consumer panic shopping, but longer-term availability challenges may also unfold. The COVID-19 pandemic threatens the accessibility of food through effects on food costs and infrastructure, including changes in food assistance distribution, public transit access, and shortages of certain products. In terms of utilization, market reports indicate widespread changes in food purchasing behaviors [14,15].

Few peer-reviewed studies so far are using empirical evidence to document actual changes in food insecurity due to COVID-19 [16]. Using statewide survey data from Vermont, a U.S. state with a predominantly rural population [17], we describe the impact of COVID-19 on household food insecurity among 3219 Vermont respondents, including their challenges and concerns related to food access, coping strategies, and use of assistance programs, and then discuss public and individual health implications of rising food insecurity. Finally, we discuss the many potential opportunities to provide resources and assistance, including primary care, to alleviate food insecurity challenges.

## 2. Materials and Methods

### 2.1. Survey Development and Recruitment

With feedback from key state-level agencies and hunger relief organizations, as well as reviews of relevant literature [3,6], we developed a survey [18] to measure food insecurity, food access challenges, and related concerns and experiences. We obtained Institutional Review Board approval from the University of Vermont (IRB protocol 00000873). Using Limesurvey [19], the instrument was piloted with 25 adults (18 years and older) from the target population. Factor analysis and Cronbach’s alpha on pilot data determined that relevant questions obtained alpha validity above 0.70 [20]. The survey ran online from 29 March to 12 April 2020. We used four methods for convenience sample recruitment: (1) paid advertisements via Front Porch Forum, a community-level listserv, which reaches approximately 2/3 of Vermont households [21]; (2) paid digital ads via Facebook to reach populations under-represented in Front Porch Forum (e.g., males, lower-income households); (3) listservs of community partners; (4) a University of Vermont press release and subsequent newspaper, radio, and television media.

**Figure A1:**
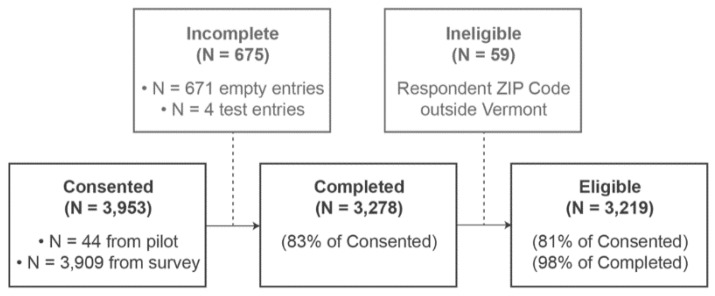
Survey consent and eligibility breakdown. Respondent surveys that were completely empty (*n* = 675) were removed from analysis. Completed responses include both partial and full surveys.

Vermont’s adult population is 506,631 [22], requiring a sample size of 2390 to achieve a 95% confidence level for a +/- 2% confidence interval. The survey had 3953 respondents, including the pilot. Respondents with ZIP Codes outside Vermont (*N* = 59) and empty responses (i.e., people who consented but did not fill in any responses, *N* = 675) were removed, leaving 3219 eligible responses (Figure A1).

Household food security status was determined based on the U.S. Department of Agriculture’s (USDA) Household Food Security Survey Module: Six-Item Short Form [23], which was adapted to ask about the time period both “in the year before the coronavirus outbreak” and “since the coronavirus outbreak.” The start of the coronavirus outbreak was set as March 8, 2020, based on the first positive COVID-19 test result in Vermont. According to established scoring procedures from the USDA food security module, respondents classified has having low (2 to 4 affirmative answers out of 6) and very low food security (5 to 6 affirmative answers) can be combined and referred to as having food insecurity [23].

In addition to measuring food security status, the survey also included additional questions related to food access challenges, use of food assistance programs, food purchasing behaviors, concerns about food access and availability, COVID-19 perceptions, and behaviors and demographics. Table A1 details the specific questions utilized in this analysis, which are primarily focused on understanding the relationship of food security status to food access challenges, use of food assistance programs, and concerns about food access and availability. Future analyses will explore other questions in the survey.

### 2.2. Statistical Analysis

To examine differences in household food insecurity during the first weeks of the COVID-19 pandemic, we created three categories of respondents: (1) households with food security (*n* = 2282, including households food secure before and since COVID-19 and households who were food insecure at some point in the year before COVID-19, but were no longer food insecure during COVID-19); (2) households with consistent food insecurity (*n* = 466, both food insecure before COVID-19 and remaining food insecure since COVID-19); (3) households with new food insecurity (*n* = 258, categorized as food secure before COVID-19, but food insecure since COVID-19). In some cases, we refer to food insecure households, which encompass both consistently food insecure households and newly food insecure households.

To determine statistically significant differences between groups we utilized Stata [24], to run Kruskal–Wallis tests, Wilcoxon rank sum tests, t-tests, and one-way analysis of variance (ANOVA) tests, depending on the distribution of the dependent variable. We used a logistical regression model to determine the factors correlated with food insecurity during the COVID-19 pandemic, with coefficients reported in odds ratios. In this model, we estimate food insecurity outcomes during COVID-19, including respondents who were classified as either consistently food insecure or newly food insecure. We used all available data to estimate effect sizes and interactions and assumed any missing data were missing at random.

## 3. Results

### 3.1. Demographic Characteristics of Respondents

Reflecting the demographic composition of Vermont [22,25,26], the majority of respondents identified as non-Hispanic White, lived in rural areas, and had a household income below $75,000 (Table 1, Table A2). Women encompassed 79% of our sample, which may be reflective of the fact that women are the dominant food shoppers in households [27].

### 3.2. Food Insecurity Prevalence

We found a nearly one-third increase (32.3%) in food insecurity prevalence (*p* < 0.001) between the year preceding the COVID-19 outbreak, when 18.8% of households (95% CI 17.38–20.13%) reported experiencing food insecurity at some point, and since the COVID-19 outbreak when the percentage rose to 24.8% (95% CI 23.27–26.35%) (Table A3). Among those experiencing food insecurity since the outbreak, 64.5% also experienced food insecurity at some point in the year prior to COVID-19, and were also food insecure since COVID-19; in comparison, 35.5% were newly food insecure. In consistently food insecure households, 59.1% exhibited very low food security since COVID-19 (marked by disrupted eating patterns and reduced intake), while 40.9% had low food security. In newly food insecure households 32.3% exhibited very low food security, while 67.7% had low food security (Table A4) since COVID-19 (*p* < 0.001).

Multivariable logit models predicted the factors contributing to higher odds of food insecurity during COVID-19 (e.g., both consistently food insecure respondents and newly food insecure respondents) (Table 2). Note that we also ran a multinomial logit model to examine whether there were statistically significant differences in newly versus consistently food insecure respondents, which there were not (Table A5). Respondents experiencing a job loss had three times greater odds of living in a household experiencing food insecurity (OR 3.06; 95% CI, 2.114–0.46), and those experiencing a furlough (OR 2.89; 95%CI, 1.864–0.49), or a loss of hours (OR 2.05; 95% CI, 1.452–0.92) also had significantly greater odds of being in a household experiencing food insecurity (*p* < 0.001). The odds of experiencing food insecurity since the COVID-19 outbreak were higher among households with children (OR 2.46; 95% CI, 1.823–0.32), while households with higher 2019 incomes had reduced odds (OR 0.56; 95% CI, 0.500–.61) (*p* < 0.001). Finally, women were 42% more likely to experience household food insecurity during COVID-19, compared to men (*p* < 0.10) (OR 1.42; 95% CI 0.9632–0.10), while a college degree (OR 0.38, 95% CI 0.290–0.50) was associated with reduced odds of household food insecurity (*p* < 0.001).

### 3.3. Food Access Challenges and Concerns

**Figure 1:**
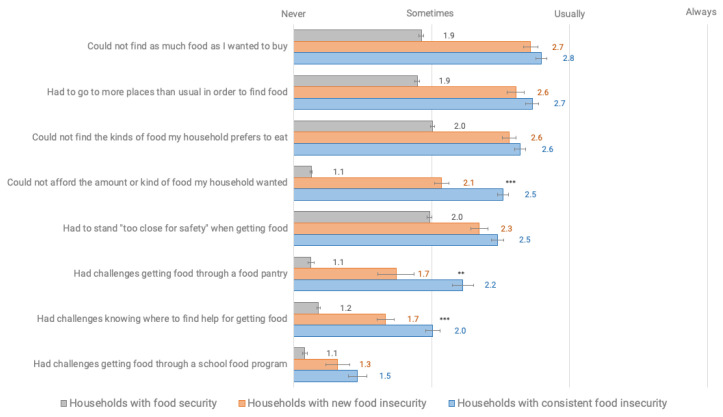
Average frequency of challenging food access situations since COVID-19 among respondents with household food security and food insecurity in a survey of Vermont households, March–April, 2020 (*p* < 0.001 for comparison among all groups). Standard errors shown with brackets. Differences between newly and consistently food insecure shown through stars (*** *p* < 0.001), ** *p* < 0.01) and in Table A6.

Respondents indicated multiple physical and economic barriers to food access during COVID-19, with respondents experiencing household food insecurity significantly more likely to express greater access, availability, and utilization challenges than respondents in food secure households (*p* < 0.001) (Figure 1, Table A6). These challenges included not finding as much or the kinds of food that someone wanted, going to more places than usual to find food, and not being able to afford the food a household wanted. Challenges also included those related to food assistance, including at food pantries and through school food programs. Consistently food insecure households had a higher average prevalence of food access challenges, as compared to those in newly food insecure households including trouble affording food (*p* < 0.001), getting food through a food pantry (*p* = 0.002), and knowing where to find help for getting food (*p* < 0.001).

**Figure 2:**
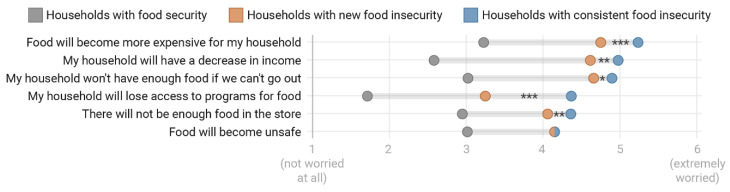
Average level of concern for potential food access situations during COVID-19 among respondents with household food security and food insecurity in a survey of Vermont households, March–April, 2020 (*p* < 0.001 for comparison among all groups). Standard errors shown with brackets. Statistically significant differences were also found between newly and consistently food insecure in all cases except for “food will become unsafe” (shown through stars, *** *p* < 0.001), ** *p* < 0.01, * *p* < 0.05) and in Table A7.

Respondents experiencing household food insecurity during COVID-19 (both newly and consistently food insecure) were significantly more likely (*p* < 0.001 comparison across all groups) to express higher levels of concern and worry about a variety of potential situations related to food access and COVID-19 (Figure 2, Table A7). These situations included potential for food to become more expensive and for households to have a decrease in income, not enough food, loss of access to food programs, and food availability and safety. As compared to newly food insecure households, consistently food insecure households were also significantly more likely to have higher levels of concern and worry about food access for all situations except for food becoming unsafe (*p* < 0.05, Table A7).

### 3.4. Coping Strategies

**Figure 3:**
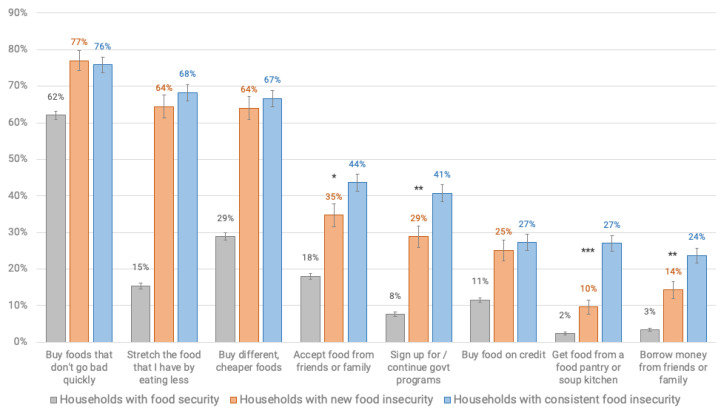
Prevalence of current coping strategies utilized by households with food security and with food insecurity during COVID-19 in a survey of Vermont households, March–April, 2020 (statistical differences among all groups *p* < 0.001). Statistical differences between newly and consistently food insecure shown through stars (*** *p* < 0.001); ** *p* < 0.01, * *p* < 0.05) and in Table A8.

Households newly and consistently experiencing food insecurity were significantly more likely (*p* < 0.001) to be implementing coping strategies related to obtaining food as compared to respondents in food secure households. These strategies included those related to disrupted eating patterns (i.e., eating less), buying different, cheaper foods, accepting food from friends and family, and utilizing government programs, credit or food pantries (Figure 3, Table A8).

Consistently food insecure households, as compared to those newly experiencing food insecurity, were also significantly more likely to currently accept food (*p* = 0.031) or borrow money from friends or family (*p* = 0.01), use a food pantry (*p* < 0.001) and use government assistance programs (*p* = 0.004), especially the Supplemental Nutrition Assistance Program (SNAP) (*p* < 0.001) (Table A8 and Table A9).

Households newly and consistently experiencing food insecurity were also significantly more likely (*p* < 0.001 across all group comparisons) to report an intention to implement these same coping strategies in the future for assistance with obtaining food during COVID-19. Among food insecure households, those with consistent food insecurity were more likely to indicate that in the future they would accept food from friends or family (*p* = 0.045), use food pantries (*p* < 0.001), government assistance programs (*p* < 0.001), and to stretch the food they have by eating less (*p* = 0.007), as compared to newly food insecure households (Table A10).

### 3.5. Desired Interventions

Compared to food secure households, new and consistently food insecure households were significantly more likely (*p* < 0.001) to find strategies to address physical or economic food access challenges helpful during COVID-19 (Table A11). These helpful strategies included extra money to help pay for food or bills, an increase in benefits of existing food assistance programs, greater trust in the safety of going to stores and food delivery, support for food delivery costs, more or different food in stores, and information about and help with food assistance programs, among others. Consistently food insecure households were also significantly more likely than those in newly food insecure households to find access to public transit, extra money for food or bills, increased benefits of food assistance programs, information about food assistance programs (all *p* < 0.001), help with administrative food assistance problems (*p* = 0.001), and support for food delivery costs (*p* = 0.033) more helpful (Table A11).

## 4. Discussion

This statewide survey in Vermont documented a statistically significant increase in food insecurity since the state’s first reported case of COVID-19 and the stay-at-home executive order (which began March 24, 2020). We demonstrate a nearly one-third increase in household food insecurity among respondents, with individuals experiencing job loss or disruption at significantly greater odds of experiencing household food insecurity since COVID-19, as compared to other demographic controls. Further, we find that the majority of consistently food insecure households and nearly one-third of newly food insecure households were classified as having very low food security, marked by disrupted eating and cutting meals or going hungry. Fully, two-thirds of Vermont respondent households with food insecurity during COVID-19 are already eating less to stretch their food. The findings indicate challenges to all food security dimensions, including economic and physical access, availability, utilization, and stability, and may have profound potential health impacts.

We further demonstrate physical and economic barriers to food access during COVID-19 and the respondents’ coping strategies in food insecure households. Previous research [10,11] suggests links between job loss and food insecurity, indicating that the profound increase in Americans experiencing job loss and disruption [28] will present acute and large-scale impacts across the population. Since Vermont unemployment claims reflect the national trend, these results likely reflect a broader U.S. phenomenon of rising food insecurity rates, evidenced by early non-peer reviewed studies [29,30]. In addition to these new economic barriers, the pandemic presents many new physical barriers for food access, reductions in public transportation, and new distribution models, and in a rural state like Vermont, a lack of income for transportation costs including fuel. In rural areas where food assistance programs, such as food pantries, are limited, closures due to illness, social distancing, or lack of volunteers may be particularly challenging. This presents opportunities to expand food pantries and support mobile pantry units, as well as encourage the expansion of programs such as fruit and vegetable prescription programs, shown to positively affect food security [31] and improve health outcomes [32]. Ultimately, this research demonstrates a need to increase food assistance programs and provide resources to remove food access barriers now, and likely in the future, during state and national economic and health emergencies.

This rise in food insecurity presents many potential health impacts. Food insecurity is negatively associated with health outcomes [5,6] and some evidence indicates it is positively associated with poor diet quality [33,34]. Further, higher rates of anxiety and mental health disorders among children and adults have been documented in food insecure households [6,33]. Indeed, survey respondents in this study experiencing household food insecurity demonstrated significantly higher rates of concern and worry about food. Disrupted eating, found in two-thirds of respondent households with food insecurity, is associated with decreased immune function and can negatively impact mental and emotional health [33]. Further research is needed to understand how food insecurity during the COVID-19 pandemic relates to diet quality, particularly if disrupted eating patterns persist and increase.

Healthcare providers can address food insecurity through simple interventions. Screening for food insecurity and providing resources now may reduce short- and long-term consequences, including the potential long-term impacts on child health outcomes associated with the duration of household food insecurity [35] and higher health care expenditures associated with food insecurity [9]. The Hunger Vital Sign, a validated two-question food insecurity screening tool based on the USDA Household Food Security Survey Module [36], can quickly determine risk for food insecurity in clinical and community settings. This tool is widely utilized, especially in pediatrics [37,38], and could be made standard in health care and other service settings during COVID-19 and beyond. Providers could refer families in need to locally available resources or to United Way, which aggregates these resources locally. However, during this heightened time of unemployment, there is also potential for government agencies, particularly those distributing unemployment benefits, to help connect families in need to available resources as well.

Importantly, this research demonstrates there are still a significant number of food insecure households which, even if aware of food assistance programs, may not use them. Low rates of seeking assistance in our results, especially among newly food insecure households, may be partly related to the stigma associated with assistance programs [39,40]. Prior research suggests that populations living outside major metropolitan areas may be more likely to use friends and family for support [41] and to see government assistance programs as a “last resort” [42]. However, with social distancing and widespread financial challenges, such personal safety nets may be eroded, and these households may be particularly vulnerable. Additional research is needed to understand the barriers to using food assistance programs, especially among those that may be newly food insecure since COVID-19.

This study suggests some of the first population-level impacts of COVID-19 and social distancing policies on food insecurity. The limitations are partly rooted in the need to rapidly administer this survey in the early days of the pandemic, to provide data that can be tracked over time. Though our respondent population matches statewide census statistics closely on many metrics, this was a convenience sample; further research is expanding these results using similar questions with representative samples across states and populations. It is worth noting that our observed overall rate of food insecurity prior to COVID-19 (18.8%) is above the most recently available state figure (11.9%) in 2018. There are potentially multiple reasons for this. First, this is likely to be due, in part, to a higher than average number of female respondents and respondents in households with children; both groups have been documented, in Vermont and elsewhere, to have elevated rates of food insecurity [43]. Second, our measurement instrument for documenting food security, the USDA 6-Item Food Security Module, includes a subjective experience domain that measures concern about household food supplies. According to the local media [44], anxiety about household food supplies preceded the Stay Home/Stay Safe order and may explain the higher than expected level of food insecurity prior to COVID-19. Further, we used an internet-based survey, given the necessity of social distancing during COVID-19 and the need for a rapid response, which may limit the capacity of some people to participate, although 81% of Vermonters do have internet plans [26]. The study’s strengths include its large sample size, early administration, population-based assessment, and survey instrument addressing the multiple dimensions of food security.

We implemented this survey in the beginning of a stay at home order and COVID-19 economic impacts. As such, it is likely that many respondents experiencing job loss or disruption had not yet received unemployment benefits and federal stimulus checks were not distributed. Future research will examine the evolution of food security impacts, and how various interventions, including the CARE Act and unemployment benefits, as well as food assistance expansion and health care screenings, may affect food insecurity outcomes as COVID-19 unfolds.

## Figures and Tables

**Table 1:** Characteristics of survey respondent individual and household demographics.

Characteristic *		Respondents (*N* = 3219)
Mean age (range) – yr		51.5 ± 15.6 (19 to 94)
Household size (range) – no.		2.7 ± 1.5 (1 to 12)
Gender – no. (%)	Female	2274 (79.4)
	Male	539 (18.8)
	Non-binary	22 (0.8)
	Transgender	13 (0.5)
	Other (self describe)	16 (0.6)
Race – no. (%)	White	2669 (96.1)
	Two or more races	73 (2.6)
	American Indian or Alaska Native	18 (0.6)
	Asian	13 (0.5)
	Black or African American	5 (0.2)
Ethnicity – no. (%)	Not Hispanic or Latino	2783 (98.4)
	Hispanic or Latino	45 (1.6)
Education level – no. (%)	Some high school (no diploma)	11 (0.4)
	High school graduate (incl. GED)	260 (9.1)
	Some college (no degree)	423 (14.8)
	Associates degree/technical school/apprenticeship	301 (10.5)
	Bachelor’s degree	962 (33.6)
	Postgraduate/professional degree	910 (31.7)
2019 Household Income – no. (%)	Less than $12,999 per year	167 (6.0)
	$13,000–$24,999 per year,	332 (11.9)
	$25,000–$49,999 per year,	672 (24.0)
	$50,000–$74,999 per year	560 (20.0)
	$75,000–$99,999 per year	442 (15.8)
	$100,000–$124,999 per year	290 (10.4)
	$125,000–$149,999 per year	141 (5.0)
	More than $150,000 per year	193 (6.9)
ZIP Code within Census Metropolitan Statistical Area – no. (%)	Yes	1149 (41.1)
	No	1649 (58.9)
Children in household – no. (%)	Yes	913 (41.9)
	No	1267 (58.1)

* Plus-minus values are means ±SD. Percentages may not total 100 because of rounding. Percentages are calculated using the number of respondents for that unique question and do not include missing data.

**Table 2:** Multivariate analysis predicting odds of food insecurity since COVID-19 (*N* = 1539).

Variable	Odds Ratio	Standard Error	*P*=	95% Confidence Interval	
Age	0.995	0.006	0.350	0.983	1.006
Race (white)	0.731	0.267	0.392	0.358	1.496
Job Loss	3.064	0.586	0.000	2.107	4.457
Furlough	2.885	0.649	0.000	1.856	4.485
Lost Hours	2.053	0.368	0.000	1.446	2.916
Female	1.422	0.283	0.077	0.963	2.100
Children	2.459	0.379	0.000	1.818	3.325
College Degree	0.380	0.055	0.000	0.286	0.506
Income	0.556	0.030	0.000	0.501	0.618
Urban Metro County	1.024	0.151	0.871	0.767	1.368

## Appendix A

**Table A1:** Complete list of variables, questions and scales used in analysis.

Variable	Question	Scale
Food Insecure	Determined based on the responses to the U.S Household Food Security Survey Module: Six-Item Short Form. These households were food insecure during COVID-19, including newly food insecure and consistently food insecure households	Binary (1 = Food Insecure, 0 = Food Secure)
Newly Food Insecure	Determined based on the responses to the U.S Household Food Security Survey Module: Six-Item Short Form. These households were classified as not food insecure during the year prior to COVID-19, but were classified as food insecure since COVID-19.	Binary (1 = Newly Food Insecure, 0 = Food Secure)
Consistently Food Insecure	Determined based on the responses to the U.S Household Food Security Survey Module: Six-Item Short Form. These households were classified as food insecure both in the year prior to COVID-19 and since COVID-19.	Binary (1 = Consistently Food Insecure, 0 = Food Secure)
Food Secure	Determined based on the responses to the U.S Household Food Security Survey Module: Six-Item Short Form. These households were not classified as food insecure during COVID-19.	Binary (1 = Food Secure, 0 = Food Insecure)
Age	In what year were you born? (age determined by subtracting birth year from 2020)	Continuous
Household size	How many people in the following age groups currently live in your household (household defined as those currently living within your household, including family and non-family members)?	Number of people (07– + ) of household members in ages 0–17, 18–65, 65 +
Children	Whether respondent indicated any children in household size	Binary
Gender	Which of the following best describes your gender identity?	Binary (Female = 1, Male = 0) *
Race (White)	What is your race? Check all that apply.	Binary (White = 1, non-white = 0)
Education	What is the highest level of formal education that you have?	Some high school = 1; High school graduate = 2; Some college = 3; Associates degree/technical school/apprenticeship = 4; Bachelor’s degree = 5; Postgraduate/professional degree = 6
College	Indication of a bachelor’s degree, postgraduate/professional degree in education	Binary (1 = College, No College = 0)
Income	Which of the following best describes your household income range in 2019 before taxes?	Less than $12,999 per year= 1; $13,000- $24,999 per year = 2; $25,000-$49,999 per year = 3; $50,000-$74,999 per year =4 $75,000- $99,999 per year = 5; $100,000- $124,99 per year = 6; $125,000-$149,999 = 7; More than $150,000 per year = 8
Urban Met Area	ZIP code, determination of ZIP code within metropolitan Burlington three county area (Chittenden, Franklin, Grand Isle)	Binary (1 = Urban, Rural = 0)
Challenge Questions	Since the coronavirus outbreak (March 8th), how often did these happen to your household?	1 = Never, 2 = Sometimes, 3 = Usually, 4 = Always, Not Applicable
	Could not afford the amount or kind of food my household wanted to buy	
	Could not find as much food as I wanted to buy (e.g., food not in store)	
	Could not find the kinds of food my household prefers to eat	
	Delivered food to a friend, neighbor, or family member	
	Had challenges getting food through a food pantry	
	Had challenges getting food through a school food program	
	Had challenges knowing where to find help for getting food	
	Had to go to more places than usual in order to find the food my household wanted	
	Had to stand “too close for safety” to other people, when getting food (less than six feet)	
Concern Questions	On a scale from 1 (not at all worried) to 6 (extremely worried), what is your level of worry for your household about the following as it relates to coronavirus.	1= not at all worried, 6= extremely worried, not applicable
	There will not be enough food in the store	
	Food will become more expensive for my household	
	Food will become unsafe	
	My household will lose access to programs that provide free food or money for food	
	My household will have a decrease in income and won’t be able to afford enough food	
	My household won’t have enough food if we have to stay at home and can’t go out at all	
Current and Future Coping Strategies	Which of the following strategies, if any, are you currently using or likely to use in the future during the coronavirus if your household has challenges affording food? Indicate both current use where applicable and future use.	Yes = 1, No = 0 for current strategies; 1 = Very Unlikely, 2 = Unlikely, 3 = Somewhat Unlikely, 4 = Somewhat Likely, 5= Likely, 6= Very Likely for future strategies
	Accept food from friends or family	
	Borrow money from friends or family	
	Buy different, cheaper foods	
	Buy food on credit	
	Buy foods that don’t go bad quickly (like pasta, beans, rice, canned foods)	
	Get food from a food pantry or soup kitchen	
	Sign up for or continue participation in a government program such as 3Squares VT or WIC or National School Lunch Program	
	Stretch the food that I have by eating less	
Helpful Strategies	What, if anything, would make it easier for your household to meet its food needs during the coronavirus pandemic?	1 = Not Helpful, 2 = Somewhat Helpful, 3 = Helpful, 4 = Very Helpful, Not Applicable
	Access to public transit or rides	
	Different hours in meal programs or stores	
	Extra money to help pay for food or bills	
	Help with administrative problems (like applying for food assistance)	
	Increase benefits of existing food assistance programs (like SNAP or WIC)	
	Information about food assistance programs or food pantries	
	More (or different) food in stores	
	More trust in safety of food delivery	
	More trust in safety of going to stores	
	Support for the cost of food delivery	

* Transgender, non-binary, and prefer to self described were not a significant portion of responses, and are reported in Table 1, but were not included in the statistical analysis because of the small sample size.

**Table A2:** Comparison of survey population and Vermont Census estimated data.

Characteristic *		Respondents (*N* = 3219)	Food Secure	Newly Food Insecure	Consistently Food Insecure
Mean age (range) – yr		51.5 ± 15.6 (19–94)	52.2 ± 15.7 (20–94)	45.4 ± 14.0 (20–85)	46.9 ± 14.2 (19–78)
Household size (range) – no.		2.7 ± 1.5 (1–12)	2.6 ± 1.3 (1–12)	3.2 ± 1.7 (1–12)	2.9 ± 1.8 (1–11)
Gender – no. (%)	Female	2274 (79.4)	1607 (78.0)	199 (85.4)	333 (81.6)
	Male	539 (18.8)	424 (20.6)	28 (12.0)	59 (14.5)
	Non-binary	22 (0.8)	14 (0.6)	2 (0.8)	6 (1.5)
	Transgender	13 (0.5)	5 (0.2)	2 (0.8)	6 (1.5)
	Other (self describe)	16 (0.6)	10 (0.4)	2 (0.8)	4 (1.0)
Race – no. (%)	White	2669 (96.1)	1939 (97.2)	224 (96.0)	366 (91.7)
	Two or more races	73 (2.6)	40 (2.0)	7 (3.0)	21 (5.0)
	American Indian or Alaska Native	18 (0.6)	8 (0.4)	0 (0.00)	6 (1.5)
	Asian	13 (0.5)	6 (0.3)	1 (0.4)	4 (1.0)
	Black or African American	5 (0.2)	2 (0.2)	1( 0.5)	2 (0.5)
Ethnicity – no. (%)	Not Hispanic or Latino	2783 (98.4)	2005 (98.5)	97.8 (0.02)	98.3 (0.02)
	Hispanic or Latino	45 (1.6)	31 (1.5)	5 (2.2)	7 (1.7)
Education level – no. (%)	Some high school (no diploma)	11 (0.4)	2 ( < 0.01)	1 (0.4)	6 (1.5)
	High school graduate (incl. GED)	260 (9.1)	118 (5.7)	34 (15.0)	90 (22.1)
	Some college (no degree)	423 (14.8)	230 (11.1)	48 (20.6)	109 (26.8)
	Associates degree/technical school/apprenticeship	301 (10.5)	193 (9.4)	25 (10.7)	65 (16.0)
	Bachelor’s degree	962 (33.6)	749 (36.3)	76 (32.6)	94 (23.1)
	Postgraduate/professional degree	910 (31.7)	771 (37.4)	49 (21.0)	43 (10.6)
2019 Household Income – no. (%)	Less than $12,999 per year	167 (6.0)	60 (3.0)	21 (9.2)	72 (17.7)
	$13,000–$24,999 per year,	332 (11.9)	147 (7.3)	37 (16.2)	131 (32.2)
	$25,000–$49,999 per year,	672 (24.0)	433 (21.5)	74 (32.5)	133 (32.7)
	$50,000–$74,999 per year	560 (20.0)	426 (21.2)	54 (23.7)	49 (12.0)
	$75,000–$99,999 per year	442 (15.8)	376 (18.7)	22 (9.6)	12 (2.9)
	$100,000–$124,999 per year	290 (10.4)	257 (12.8)	13 (5.7)	7 (1.7)
	$125,000–$149,999 per year	141 (5.0)	126 (6.3)	4 (1.8)	1 (0.2)
	More than $150,000 per year	193 (6.9)	181 (9.0)	3 (1.3)	2 (0.4)
ZIP Code within Census Metropolitan Statistical Area – no. (%)	Yes	1149 (41.1)	1156 (57.2)	141 (62.4)	247 (61.2)
	No	1649 (58.9)	864 (42.8)	85 (37.6)	153 (38.3)
Children in household – no. (%)	Yes	913 (41.9)	590 (37.7)	118 (62.4)	173 (53.6)
	No	1267 (58.1)	975 (62.3)	71 (37.6)	150 (46.4)

* Plus-minus values are means ±SD. Percentages may not total 100 because of rounding. Percentages are calculated using the number of respondents for that unique question and do not include missing data.

**Table A3:** Results of a two-sided t-test for difference in food insecurity rates.

Variable	*n*=	Mean	Std Error	Std. Dev.	95% Confidence Interval	
Food Insecure in Previous 12 months	3086	0.188	0.007	0.390	0.174	0.197
Food Insecure Since COVID-19	3028	0.248	0.008	0.432	0.233	0.259
*p* < 0.001						

**Table A4:** Prevalence of food insecurity among food insecure respondents prior to and since COVID-19 by USDA categorization.

	In the Year Prior to COVID-19	Since COVID-19	
Aggregate Score *	Consistently Food Insecure	Consistently Food Insecure	Newly Food Insecure
2	21.17%	15.30%	30.04%
3	15.51%	15.30%	23.95%
4	14.05%	10.27%	13.69%
5	13.41%	17.61%	14.83%
6	35.85%	41.51%	17.49%
Low Food Security	50.73%	40.87%	67.68%
Very Low Food Security	49.26%	59.12%	32.32%

* Classification of 2 or higher on the USDA scale ranging from 0 to 6 indicates food insecurity. Individuals with 2–4 classification are considered to have low food security, while individuals with scores ranging from 4–6 are considered to have very low food security. Pearson chi2(4) = 56.9921 *p* < 0.001.

**Table A5:** Multinomial logit regression model results for comparing newly food insecure to consistently food insecure households predicting food insecurity since COVID-19.

Newly Food Insecure Respondents					
**Variable**	**Coefficient**	**Standard Error**	***P*** **=**	**95% Confidence Interval**	
Age	−0.010	0.008	0.235	−0.025	0.006
Race (white)	−0.415	0.456	0.363	−1.310	0.479
Job Loss	1.423	0.249	0.000	0.935	1.910
Furlough	1.016	0.317	0.001	0.395	1.637
Lost Hours	0.802	0.244	0.001	0.323	1.281
Female	0.426	0.280	0.128	−0.122	0.975
Children	0.981	0.209	0.000	0.571	1.391
College Degree	−0.567	0.200	0.005	−0.958	−0.176
Income	−0.398	0.068	0.000	−0.531	−0.265
Urban Metro County	−0.134	0.199	0.499	−0.523	0.255
Consistently Food Insecure Respondents					
**Variable**	**Coefficient**	**Standard Error**	***P*** **=**	**95% Confidence Interval**	
Age	−0.003	0.007	0.656	−0.016	0.010
Race (white)	−0.231	0.441	0.600	−1.094	0.633
Job Loss	0.885	0.231	0.000	0.433	1.337
Furlough	1.075	0.264	0.000	0.558	1.591
Lost Hours	0.636	0.219	0.004	0.206	1.065
Female	0.337	0.239	0.160	−0.133	0.806
Children	0.838	0.187	0.000	0.472	1.204
College Degree	−1.224	0.176	0.000	−1.568	−0.879
Income	−0.760	0.070	0.000	−0.897	−0.622
Urban Metro County	0.136	0.177	0.443	−0.212	0.484

The base outcome comparison is food secure households. We find no significantly differences between the factors predicting food insecurity since COVID-19 for newly or consistently food insecure households, so report the combined results of a multivariable logit model in the main results of both newly and consistently food insecure households together.

**Table A6:** Prevalence of challenges during COVID-19.

	Food Secure		Newly Food Insecure		Consistently Food Insecure		*P* Value	
Situation	Mean	95% CI	Mean	95% CI	Mean	95% CI	All Groups	New and Consistently Food Insecure
Could not afford the amount or kind of food my household wanted to buy	1.13	1.11–1.14	2.07	1.97–2.18	2.52	2.44–2.60	<0.001	<0.001
Could not find as much food as I wanted to buy (e.g., food not in store)	1.92	1.89 = 1.96	2.72	2.61–2.82	2.79	2.71–2.88	<0.001	0.246
Could not find the kinds of food my household prefers to eat	2.01	1.97–2.04	2.56	2.47–2.66	2.64	2.56–2.72	<0.001	0.232
Had challenges getting food through a food pantry	1.12	1.08–1.17	1.74	1.48–2.00	2.23	2.08–2.38	<0.001	0.002
Had challenges getting food through a school food program	1.08	1.04–1.11	1.32	1.15–1.49	1.46	1.33–1.59	<0.001	0.081
Had challenges knowing where to find help for getting food	1.18	1.15–1.20	1.66	1.54–1.79	2.01	1.91–2.11	<0.001	<0.001
Had to go to more places than usual in order to find the food my household wanted	1.89	1.86–1.93	2.61	2.49–2.73	2.73	2.64–2.82	<0.001	0.123
Had to stand “too close for safety” to other people, when getting food (less than six feet)	1.99	1.95–2.02	2.34	2.22–2.47	2.48	2.39–2.57	<0.001	0.096

*P* values among all groups conducted using Kruskal Wallis Test. *P* values among new and consistently food insecure conducted using Wilcoxon Rank Sum tests with Porder Exact *P* values.

**Table A7:** Average level of concern for food access among three groups.

	Food Secure		Newly Food Insecure		Consistently Food Insecure		*P* Value	
Question	Mean	95% CI	Mean	95% CI	Mean	95% CI	All Groups	New and Consistently Food Insecure
There will not be enough food in the store	2.95	2.89–3.00	4.07	3.90–4.24	4.35	4.23–4.48	<0.001	0.007
Food will become more expensive for my household	3.22	3.16- 3.29	4.74	4.59–4.90	5.23	5.13–5.32	<0.001	<0.001
Food will become unsafe	3.02	2.95–3.08	4.14	3.95–4.34	4.13	3.98–4.28	<0.001	0.960
My household will lose access to programs that provide free food or money for food	1.69	1.59–1.79	3.23	2.91–3.56	4.39	4.19–4.59	<0.001	<0.001
My household will have a decrease in income and won’t be able to afford enough food	2.57	2.50–2.65	4.61	4.42–4.79	4.98	4.84–5.11	<0.001	0.003
My household won’t have enough food if we have to stay at home and can’t go out at all	3.02	2.95–3.09	4.64	4.45–4.82	4.90	4.77–5.04	<0.001	0.010

*P* values among all groups conducted using Kruskal Wallis Test. *P* values among new and consistently food insecure conducted using Wilcoxon Rank Sum tests with Porder Exact *P* values.

**Table A8:** Use of current coping strategies to increase food access during COVID-19.

	Food Secure		Newly Food Insecure		Consistently Food Insecure		*P* Value	
Strategy	Mean	95% CI	Mean	95% CI	Mean	95% CI	All Groups	New and Consistently Food Insecure
Accept food from friends or family	0.18	0.16–0.20	0.35	0.29–0.41	0.44	0.39–0.48	<0.001	0.031
Borrow money from friends or family	0.03	0.03–0.04	0.14	0.10–0.19	0.24	0.20–0.28	<0.001	0.004
Buy different, cheaper foods	0.29	0.27–0.31	0.64	0.58–0.70	0.67	0.62–0.71	<0.001	0.559
Buy food on credit	0.11	0.10–0.13	0.25	0.20–0.31	0.27	0.23–0.32	<0.001	0.613
Buy foods that don’t go bad quickly (like pasta, beans, rice, canned foods)	0.62	0.60–0.64	0.77	0.72–0.82	0.76	0.72–0.80	<0.001	0.813
Get food from a food pantry or soup kitchen	0.02	0.02–0.03	0.10	0.06–0.13	0.27	0.23–0.31	<0.001	0.000
Sign up for or continue participation in a government program such as 3Squares VT or WIC or National School Lunch Program	0.08	0.06–0.09	0.29	0.23–0.35	0.41	0.36–0.45	<0.001	0.003
Stretch the food that I have by eating less	0.15	0.14–0.17	0.64	0.58–0.71	0.68	0.64–0.73	<0.001	0.360

*P* values among all groups conducted using Kruskal Wallis Test. *P* values among new and consistently food insecure conducted using Wilcoxon Rank Sum tests with Porder Exact *P* values.

**Table A9:** Use of food assistance programs during COVID-19 among households experiencing food insecurity.

	Newly Food Insecure		Consistently Food Insecure		*P* Value
**Program**	**Mean**	**95% CI**	**Mean**	**95% CI**	
Meals on Wheels	0.01	0.00–0.02	0.02	0.01–0.04	0.207
SNAP	0.11	0.08–0.15	0.28	0.23–0.31	<0.001
WIC	0.11	0.07–0.14	0.12	0.09–0.14	0.815
Food Pantry	0.08	0.04–0.11	0.21	0.17–0.25	<0.001

*P* values determined using Wilcoxon Rank Sum with Porder Exact *P* Values.

**Table A10:** Likely use of future coping strategies for food access during COVID-19.

	Food Secure		Newly Food Insecure		Consistently Food Insecure		*P* Value	
Strategy	Mean	95% CI	Mean	95% CI	Mean	95% CI	All Groups	New and Consistently Food Insecure
Accept food from friends or family	2.76	2.69–2.83	3.45	3.25–3.66	3.69	3.53–3.85	<0.0001	0.045
Borrow money from friends or family	1.97	1.91–2.03	2.78	2.57–2.99	2.78	2.60–2.95	<0.0001	0.666
Buy different, cheaper foods	3.80	3.72–3.87	4.80	4.64–4.96	4.86	4.72–4.99	<0.0001	0.189
Buy food on credit	2.45	2.37–2.52	3.18	2.94–3.42	3.09	2.89–3.29	<0.0001	0.425
Buy foods that don’t go bad quickly (like pasta, beans, rice, canned foods)	4.90	4.84–4.96	5.20	5.06–5.34	5.19	5.07–5.31	<0.0001	0.495
Get food from a food pantry or soup kitchen	1.75	1.70–1.81	2.86	2.66–3.06	3.57	3.39–3.76	<0.0001	0.000
Sign up for or continue participation in a government program such as 3Squares VT or WIC or National School Lunch Program	1.90	1.84–1.97	3.24	2.97–3.50	4.10	3.90–4.29	<0.0001	0.000
Stretch the food that I have by eating less	2.99	2.92–3.07	4.68	4.52–4.85	4.93	4.80–5.05	<0.0001	0.007

*P* values among all groups conducted using Kruskal Wallis Test. *P* values among new and consistently food insecure conducted using Wilcoxon Rank Sum tests with Porder Exact *P* values.

**Table A11:** Perceived helpful strategies to assist households with meeting food needs during COVID-19.

	Food Secure		Newly Food Insecure		Consistently Food Insecure		*P* Value	
Strategy	Mean	95% CI	Mean	95% CI	Mean	95% CI	All Groups	New and Consistently Food Insecure
Access to public transit or rides	1.20	1.16–1.24	1.25	1.12–1.37	1.80	1.64–19.6	<0.001	<0.001
Different hours in meal programs or stores	1.79	1.74–1.85	2.04	1.87–2.20	2.22	2.10–2.35	<0.001	0.101
Extra money to help pay for food or bills	2.35	2.29–2.42	3.30	3.18–3.42	3.68	3.62–3.74	<0.001	<0.001
Help with administrative problems (like applying for food assistance)	1.41	1.34–1.48	2.16	1.95–2.37	2.58	2.44–2.73	<0.001	0.001
Increase benefits of existing food assistance programs (like SNAP or WIC)	1.80	1.71–1.89	2.88	2.67–3.08	3.51	3.40–3.61	<0.001	<0.001
Information about food assistance programs or food pantries	1.60	1.53–1.68	2.35	2.16–2.53	2.77	2.65–2.89	<0.001	<0.001
More (or different) food in stores	2.77	2.73–2.82	3.20	3.09–3.31	3.27	3.18–3.36	<0.001	0.172
More trust in safety of food delivery	2.78	2.73–2.84	3.14	3.00–3.27	3.20	3.10–3.30	<0.001	0.369
More trust in safety of going to stores	3.23	3.19–3.27	3.53	3.43–3.62	3.49	3.41–3.56	<0.001	0.762
Support for the cost of food delivery	2.33	2.26–2.40	3.09	2.95–3.23	3.26	3.15–3.36	<0.001	0.015

*P* values among all groups conducted using Kruskal Wallis Test. *P* values among new and consistently food insecure conducted using Wilcoxon Rank Sum tests with Porder Exact *P* values.
